# Efficient transformation of the isolated microspores of Chinese cabbage (*Brassica rapa* L*. ssp. pekinensis*) by particle bombardment

**DOI:** 10.1186/s13007-024-01134-1

**Published:** 2024-01-30

**Authors:** Yujia Liu, Shujiang Zhang, Shifan Zhang, Hui Zhang, Guoliang Li, Rifei Sun, Fei Li

**Affiliations:** Institute of Vegetables and Flowers, Chinese Academy of Agricultural Sciences, Zhongguancun, Nandajie No. 12, Haidian District, Beijing, 100081 People’s Republic of China

**Keywords:** Chinese cabbage, Microspore, Particle bombardment, Transformation

## Abstract

**Background:**

The low efficiency of genetic transformation in Chinese cabbage (*Brassica rapa* L*. ssp. pekinensis*) is the key problem affecting functional verification. Particle bombardment is a widely used method along with the *Agrobacterium*-mediated method. As a physical means, it has almost no restrictions on the type of host and a wide range of receptor types, which largely avoids the restriction of explants. The bombardment parameters, which include the number of bombardments, the bombardment pressure, and the bombardment distance, may affect the microspores' genetic transformation efficiency.

**Results:**

The transformation efficiency was improved using the particle bombardment method under the combination of bombardment shot times (3, 4, 5) × bombardment pressure (900, 1100, 1350 psi) × bombardment distance (3, 6, 9 cm). The average viability of microspores in the treatment group ranged from 74.76 to 88.55%, while the control group was 88.09%. When the number of shot times was 4, the number of embryos incubated in the treatment group ranged from 16 to 236 per dish, and the control group had 117 embryos per dish. When the bombardment parameters of the biolistic method were 4 shot times—1350 psi—3 cm, 4 times—1100 psi—3 cm, and 4 times—900 psi—3 cm, they had high transient expression efficiency, and the average number of transformed microspores was 21.67, 11.67, and 11.67 per dish (3.5 mL), respectively. When the bombardment parameters were 4 times, 900 psi, and 6 cm, the highest genetically transformed embryos were obtained, and the transformation efficiency reached 10.82%.

**Conclusion:**

A new genetic transformation system with proper parameters for Chinese cabbage microspores was established using particle bombardment. This proper transformation system could provide a useful tool for the improvement of cultivar quality and the investigation of functional genes in Chinese cabbage.

**Supplementary Information:**

The online version contains supplementary material available at 10.1186/s13007-024-01134-1.

## Background

Chinese cabbage (*Brassica rapa* L*. ssp. pekinensis*) is one of the most important *Brassica rapa* subspecies, a significant vegetable that is widely cultured in China. In the last few decades, genetic approaches, including transformation technology, have been utilized in the genus *Brassica*, resulting in several agricultural and economically important benefits.

The *Agrobacterium*-mediated method has been widely used as the main mediating method in transgenic technology. In plant genetic engineering research, 85% of genetic transformations are successfully achieved by *Agrobacterium*-mediated methods [1]. Using transgenic technology, plants with resistance to herbicides, insects, and diseases have been successfully obtained, and plants with excellent traits have been produced to meet the needs of human beings for plant characteristics [2–7].

Genomic sequencing of Chinese cabbage as a model for the *B. rapa* A genome has facilitated the genetic identification and cloning of candidate genes governing traits [8]. However, verification of gene functions revealing the genetic mechanisms of trait formation has remained stagnant due to the lack of an efficient genetic transformation system. To a large extent, transformation efficiency is dependent on the genetic background of the *B. rapa* subspecies [9, 10]. *Brassica rapa* is widely known to be the most recalcitrant species for genetic transformation among *Brassica* species.

The regeneration frequency of *Brassica* crops varies greatly among different species and varieties and is affected by many factors [9, 11]. Common Chinese cabbage transgenic technology methods include the *Agrobacterium*-mediated method, and the conversion efficiency is in the range of 1.2–10.83% [10, 12–15]. The genetic transformation of Chinese cabbage by the *Agrobacterium*-mediated method is still limited due to the difficulty of tissue regeneration and the great influence of genotype on genetic transformation [16, 17]. Therefore, the development of a new transformation method is highly desirable.

With the development of *Brassica* crop-isolated microspore culture technology, the construction of a genetic transformation system using microspores as new explants has attracted increasing attention. Isolated microspore culture has a series of advantages, such as haploidy, a high embryogenesis rate, and single cell culture, and has gradually become one of the important means in Chinese cabbage breeding [18–21]. In 1982, *Brassica napus* was successfully cultivated by microspores, and microspore culture technology has been developed successively [22, 23].

In addition to the *Agrobacterium*-mediated method, the particle bombardment method appeared for the first time in 1987 [24–26]. It is essentially a physical process and is not limited by the receptor genotype [27, 28]. It is widely used in many plant species, such as onion, barley, bitter melon, maize, and rice [29–35]. Genetic transformation of chloroplasts, mitochondria, and nuclei of difficult-to-transform crop species can be achieved using gene gun-mediated methods [26, 36, 37]. There are many factors affecting the genetic transformation efficiency of biolistic methods, which can be divided into physical parameters, environmental parameters, and biological parameters [26]. Gold particles have higher conversion efficiency than tungsten [28, 38, 39]. The particle bombardment method combined with microspore genetic transformation has been successful in wheat and cabbage [40–42].

In this study, microspores of Chinese cabbage were used as the genetic transformation receptor, and a gene gun was used as the genetic transformation method to optimize the bombardment parameters: bombardment times, bombardment pressure, and bombardment distance. After bombardment, the changes in microspore viability and embryogenic ability were measured, the transformation efficiency of microspores and embryos were identified, and the proper genetic transformation parameters were screened. This study aimed to construct an efficient and stable new genetic transformation system and promote the widespread application of Chinese cabbage transgenic technology.

## Materials and methods

### Plant materials and growth conditions

Double haploid lines (DH) 2,014,003 (early heading Chinese cabbage), 2,014,011 (early heading Chinese cabbage), 1,900,502 (mid-late ripening heading Chinese cabbage), and 1,900,536 (mid-late ripening heading Chinese cabbage) were cultivated at the south farm greenhouse at a room temperature at the Institute of Vegetables and Flowers, Chinese Academy of Agricultural Sciences, Beijing, China.

### Vector preparation

The vector used for optimizing the particle bombardment system in Chinese cabbage was pBI121, which contained the CaMV 35S promoter and GUS screening gene, and was purchased from Beijing Zhuang Meng International Biological Gene Technology Co., Ltd. (Beijing, China). The vector pHSE401-mCherry contained the red fluorescence label promoted by mCherry and was a CRISPR/Cas9 vector. The pCAMBIA2300 vector contained the insert section of the *Dark_Pur* gene, with the CaMV35S promoter, eGFP, and Kan labels [43].

The Easy Pure Plasmid MiniPrep Kit (purchased from Transgene) was used to extract Plasmid DNA according to the manufacturer’s instructions.

### Extraction of Chinese cabbage microspores

Chinese cabbage flower buds with diameters between 2 and 3 mm were chosen. The buds were sterilized and placed in a shake pipe with B5 medium [44]. An 11-s treatment was performed at 3000 rpm amplitude using a crusher (TOMY, Micro SmashTM MS-100) to obtain the Chinese cabbage microspores. A funnel was used to filter impurities and obtain pure microspores. The suspension was centrifuged at 1500 rpm for 4 min, and purified microspores were obtained after supernatant removal. The NLN-13 medium was added to the microspores at a final concentration of 10^5^–10^6^ microspores/ml [44]. The Chinese cabbage microspore suspension was cultivated at 33 °C to induce heat shock in the dark for 24 h. The microspores of Chinese cabbage were collected from 25 ml suspensions with a concentrations ranging from 10^5^–10^6^/ml as an experimental treatment. All materials must be sterilized.

### Gold-DNA particle preparation and bombardment

Three milligrams of gold particles (Bio-Rad) with a diameter of 0.6 μm were weighed and placed in a sterile centrifuge tube. One milliliter of absolute ethyl alcohol was added to the gold particles, and the suspension was swirled. The supernatant of the gold particles was centrifuged and removed, and the previous steps were repeated twice. One milliliter of sterile ddH_2_O was added to the gold particle sediment, and the suspension was swirled again. The centrifuge tube was centrifuged at 3000 rpm for 10 s, and the supernatant was removed. Then, 50 μl of sterile ddH_2_O was added to a final concentration of 60 mg/ml.

The following reagents were added in turn to a 50-μl gold suspension: 5 μl of prepared plasmid DNA (1000 ng/μl), 50 μl of CaCl_2_ (2.5 M, Cat#C8370 Solarbio), and 20 μl of protamine (1 mg/ml, Lot#SLBR3313V, Sigma-Aldrich). The suspension was swirled and then centrifuged at 3000 rpm for 90 s. The supernatant was removed, and 250 μl absolute ethyl alcohol was added to the gold-DNA particle sediment. The previous step was repeated, and 60 µl of anhydrous ethanol was added for a constant volume. Each shot used 10 μl gold-DNA particle.

### Morphological comparison of Chinese cabbage microspores and gold particles and verification of the effectiveness of gold particle DNA bullets

The effectiveness of plasmid DNA GUS-gold particle bullets was verified by bombarding Chinese cabbage leaves with a gene gun, and the parameters were 1 time, 1100 psi, and 6 cm. After 72 h of culture, the Chinese cabbage leaves were stained with GUS staining solution to test bullet effectiveness (G3060-100 ml, Solarbio).

### Viability of microspore cells and regeneration ability of Chinese cabbage after particle bombardment

Three Chinese cabbage microspore samples (50 μl for each treatment) were collected randomly and applied to glass slides. Acetate magenta staining solution was added to the Chinese cabbage microspores for 15 min. The number of viable red microspores and the total number of microspores were counted in the field of view under a microscope at 10x. The following formula was used to calculate the viability of microspores after bombardment: microspore activity (%) = number of viable microspores/total number of microspores × 100%. The same formula was used to calculate the microspores that were not shot (CK). SPSS version 26 (SPSS, Chicago, IL, USA) software for Windows was used to determine significant differences between the treatment group and CK. The experiment was designed with two technical replicates and three biological replicates. The number of embryos developed per experimental treatment was counted. The number of embryos not shot by a gene gun constituted the CK.

### Optimization of the gene gun-mediated transformation of Chinese cabbage microspores

Three variables were designed to optimize the system of particle bombardment of Chinese cabbage microspores: bombardment time (3, 4, 5), pressure of bombardment (900, 1100, 1350 psi), and distance of bombardment (3, 6, 9 cm). Each operation was repeated with two technical replicates and three biological replicates. Twenty-five milliliters of Chinese cabbage microspores, which were heated at 33 °C in the dark for 24 h, were collected after centrifuging at 1500 rpm for 2 min and placed in the center of a 10 × 10 cm plastic Petri dish. The microspores were re-suspended with NLN-13 medium at a concentration of 10^5^–10^6^ microspores/ml and cultivated at 25 °C in the dark after being shot.

### Transformation efficiency of Chinese cabbage microspores by particle bombardment

After 72 h of cultivation, the microspores were collected to detect the transformation efficiency. Chinese cabbage microspore samples of petri dishes for each treatment were collected in a 50 ml centrifuge tube, and the supernatant was removed. The GUS solution (G3060-100 ml, Solarbio) was added to the microspores cells to incubate for 12–24 h at 37 °C. The transformed microspores that expressed dark blue were counted using an inverted microscope (AX10 Vert.A1, Zeiss, Germany). The microspores that were not shot by the gene gun were treated as CK.

After 15 d of cultivation, the embryos were incubated with the GUS solution. The number of blue embryos and the total number of embryos were counted to calculate the transformation efficiency. The embryos generated from microspores that were not shot were also incubated in the GUS solution as CK. The following formula was used to calculate the transformation efficiency:

$${\text{Transformation\,efficiency }}\left( \% \right)\, = \,{\text{the\,number\,of\,blue\,embryos}}/{\text{the\,total\,number\,of\,embryos}}\, \times \,100\% .$$

### DNA extraction and PCR identification from transgenic Chinese cabbage

The leaves of plants generated from the bombardment of Chinese cabbage microspores were collected. The CTAB method was used to extract the DNA of the transformed plants generated from Chinese cabbage microspores. The primers linked to the GUS label were designed to detect positive plants (Table 1). At the same time, GUS histochemical staining was performed on each plant sample. Plants with blue GUS histochemical staining and positive PCR identification were selected for self-pollination, and T1 generation plants were harvested. GUS histochemical staining and PCR identification were performed on T1 generation plants.

**Table 1:** PCR primers for identifying positive plants

Primer name	Primer sequence (5′–3′)
GUS_F	CAACGAACTGAACTGGCAGA
GUS_R	GAGCGTCGCAGAACATTACA

### Fluorescence observation of transformed microspores in Chinese cabbage

The vector pHSE401-mCherry was used to shoot the Chinese cabbage microspores. Due to the red fluorescence expressed by the mCherry label, transformed microspores were easily observed. The microspores that were shot after 72 h were collected, and an inverted fluorescence microscope (AX10 Vert.A1, Zeiss, Germany) was used to detect the red microspores. The microspores that were not shot (CK) were also observed using a microscope.

## Results

### Particle bombardment-mediated genetic transformation of Chinese cabbage microspores

**Fig. 1:**
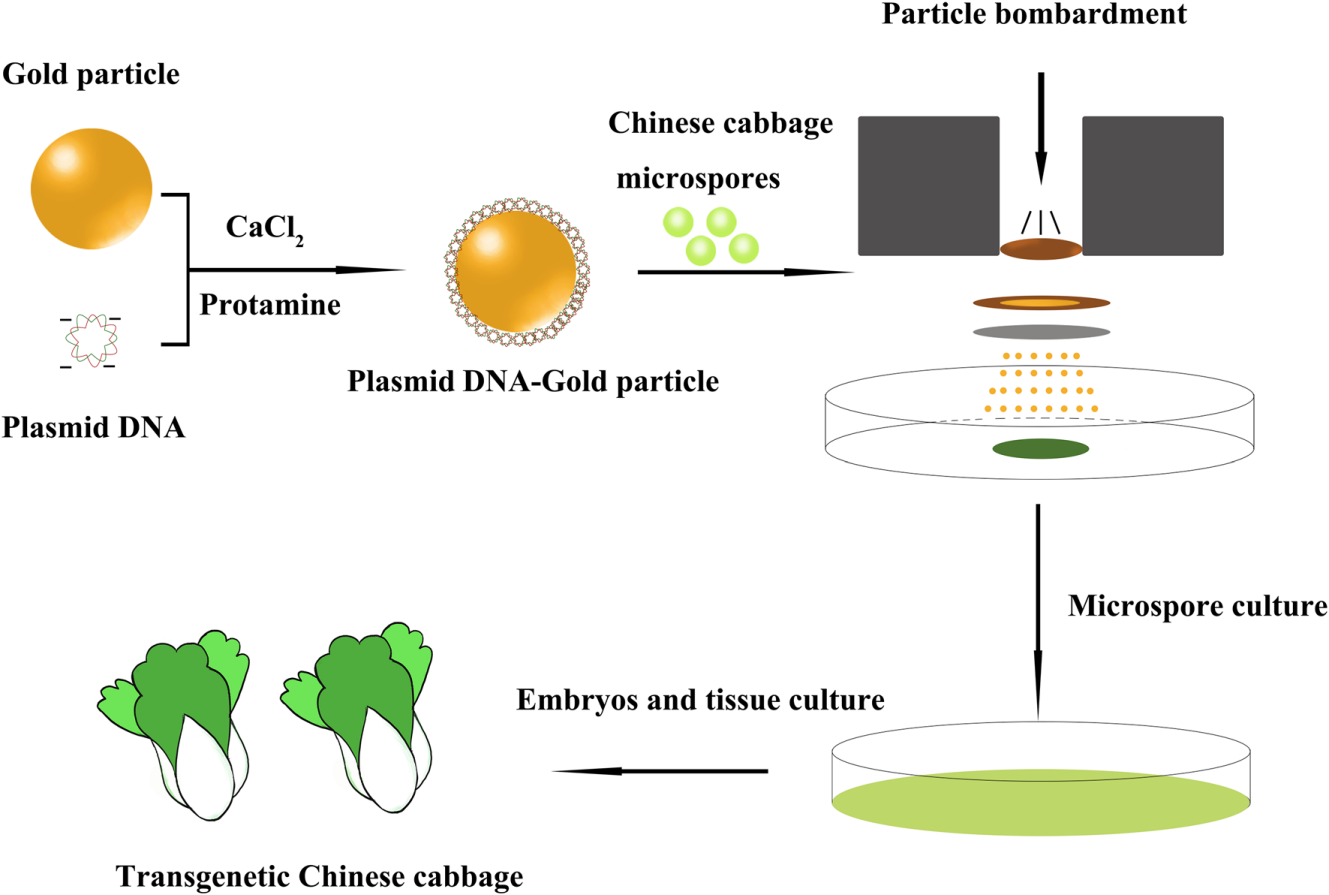
Schematic diagram of particle bombardment-mediated genetic transformation of Chinese cabbage microspores

The process of genetic transformation of Chinese cabbage microspores by the particle bombardment method consisted of the following steps. First, plasmid DNA-gold particle bullets were prepared. Under the coating of CaCl_2_ and Protamine, plasmid DNA was attached to the surface of gold particles. With the help of the impact force of a suitable exogenous high-pressure helium gas, the plasmid DNA-gold particle bullets dotted on the surface of the carrier film were launched into the interior of the Chinese cabbage microspores so that the exogenous DNA could be transferred to the Chinese cabbage microspores (Fig. 1). The transformed microspores were collected and cultured in suspension. After 15 days, embryoid bodies that developed from the microspores appeared. The embryoid bodies were dedifferentiated and redifferentiated to obtain Chinese cabbage tissue culture seedlings. Because Chinese cabbage microspores are single cells and the nucleus can naturally double to become a diploid, it is easy to obtain Chinese cabbage DH lines through the microspore culture method; that is, the theoretically obtained transformed plants are DH lines. Thus, the generation of transgenic homozygous plants can be achieved.

### Comparison between gold particles and Chinese cabbage microspores, and the validity of gold-DNA particles

**Fig. 2:**
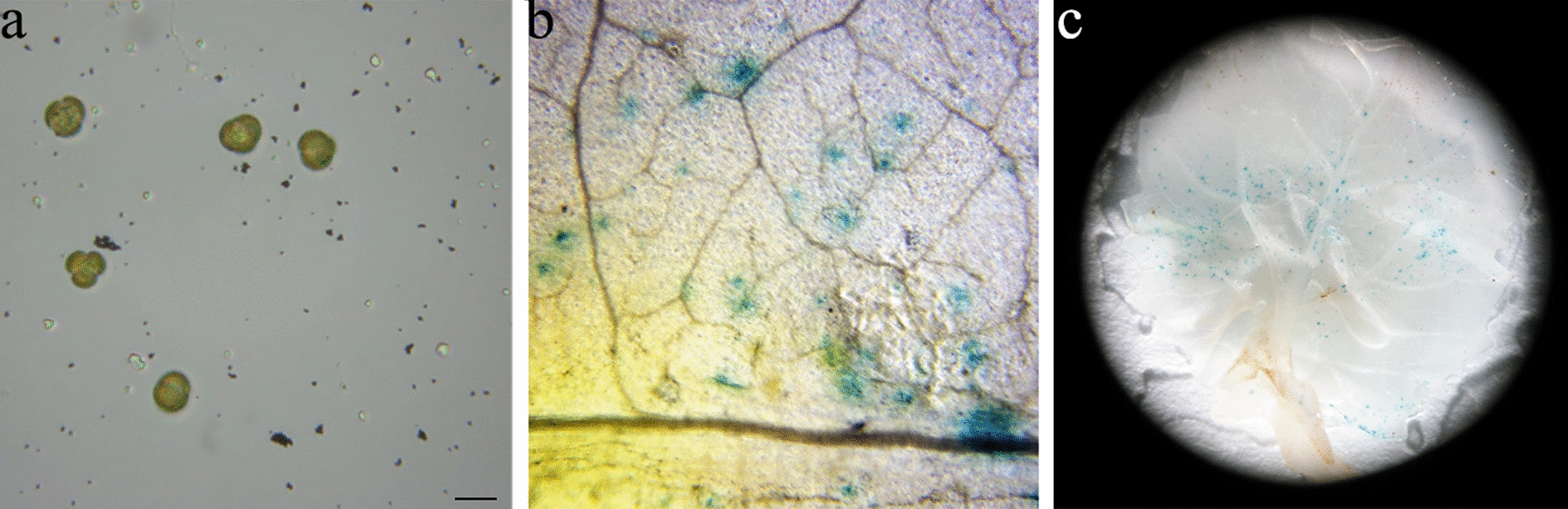
Gold particles compared with Chinese cabbage microspores and gold-DNA effectiveness observed on Chinese cabbage leaves. **a** Comparison of the size of gold-DNA particles and Chinese cabbage microspores, the scale bar is 20 μm. **b** Microscopic observation of the GUS staining of leaves after bombardment with a gene gun at 5 × magnification, where the blue spot represents the successful expression of the GUS gene. **c** Stereoscope observation of the GUS staining of leaves after bombardment with a gene gun

After bombardment, the microspores were observed using a microscope. Figure 2a indicates that the gold particles are 0.6 μm and the diameter of Chinese cabbage microspores is approximately 20 μm. The diameter of the gold-DNA particle was smaller than the microspore and germination hole, and the difference was significant. The gold-DNA particle could easily cross the microspore cell wall into the inner part of the microspore or cross the germination hole into the microspore. This provides the feasibility of genetic transformation by bombarding Chinese cabbage microspores using a biolistic method.

Before bombarding Chinese cabbage microspores with a gene gun, the effectiveness of gold-DNA particle bullets was verified by bombarding Chinese cabbage leaves using a gene gun. As shown in Fig. 2b, c, evenly distributed blue spots of GUS staining were detected on the surface of the leaves after bombardment with the gene gun, demonstrating the effectiveness of gold-DNA particles. The gold-DNA particles could be used for the subsequent genetic transformation of Chinese cabbage microspores.

### There is little effect on microspore viability, embryo emergence and regeneration after particle bombardment

**Fig. 3:**
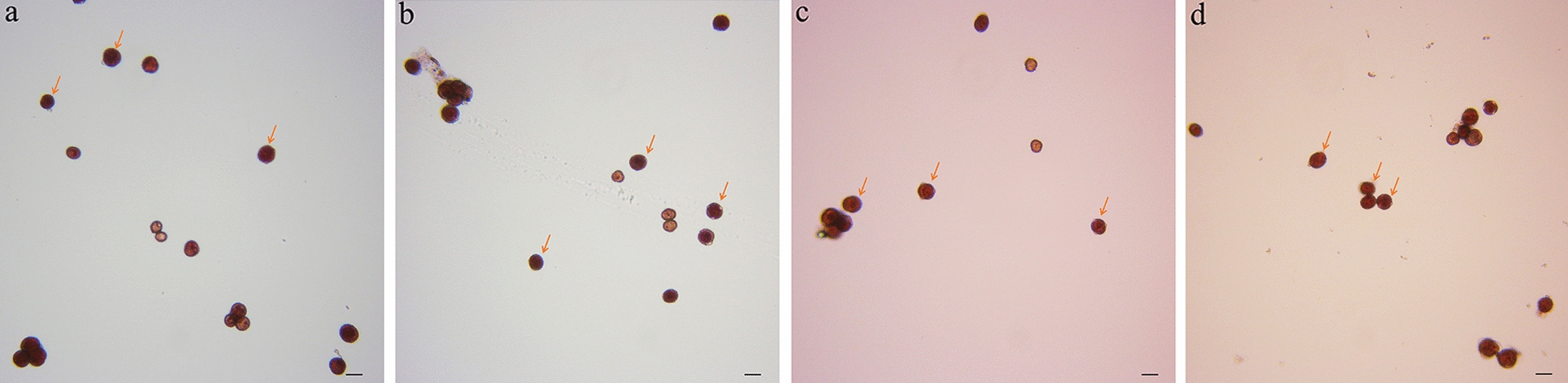
Viability of microspores after particle bombardment (72 h) observed using a microscope at 20 ×. **a** The viability of microspores under bombardment parameters of 4 times—1100 psi—3 cm. **b** The viability of microspores under bombardment parameters 4 times—1100 psi—6 cm. **c** The viability of microspores with bombardment parameters of 4 times—1100 psi—9 cm. **d** The viability of microspores that were not bombarded. Living microspores are indicated by the arrow in the figure. The scale bar is 20 μm

The viable microspores and the total number of microspores were counted, and microspore viability was calculated. As shown in Table 2, there were no significant differences among the treatments, and there was no significant difference between the treatment and CK 3 d after bombardment. Additionally, Fig. 3 and Additional file 1: Fig. S1 show that the viability of the microspores that were shot using the gene gun was the same as in the CK.

**Table 2:** Viability of Chinese cabbage microspores

Bombardment times	Bombardment pressure (psi)	Bombardment distance (cm)	Average viability of the microspore (%)	Number
3	900	3	84.61a	1
		6	85.46a	2
		9	88.55a	3
	1100	3	86.11a	4
		6	85.87a	5
		9	87.65a	6
	1350	3	81.03a	7
		6	85.54a	8
		9	78.29a	9
4	900	3	82.16a	10
		6	80.95a	11
		9	79.38a	12
	1100	3	81.28a	13
		6	84.09a	14
		9	86.61a	15
	1350	3	74.76a	16
		6	85.31a	17
		9	82.98a	18
5	900	3	78.43a	19
		6	83.11a	20
		9	82.33a	21
	1100	3	85.75a	22
		6	87.63a	23
		9	85.83a	24
	1350	3	80.91a	25
		6	81.20a	26
		9	87.07a	27
CK			88.09a	

The different lowercase letters in the same column indicate significant differences at P < 0.05 by Tamhane's new complex range test

**Fig. 4:**
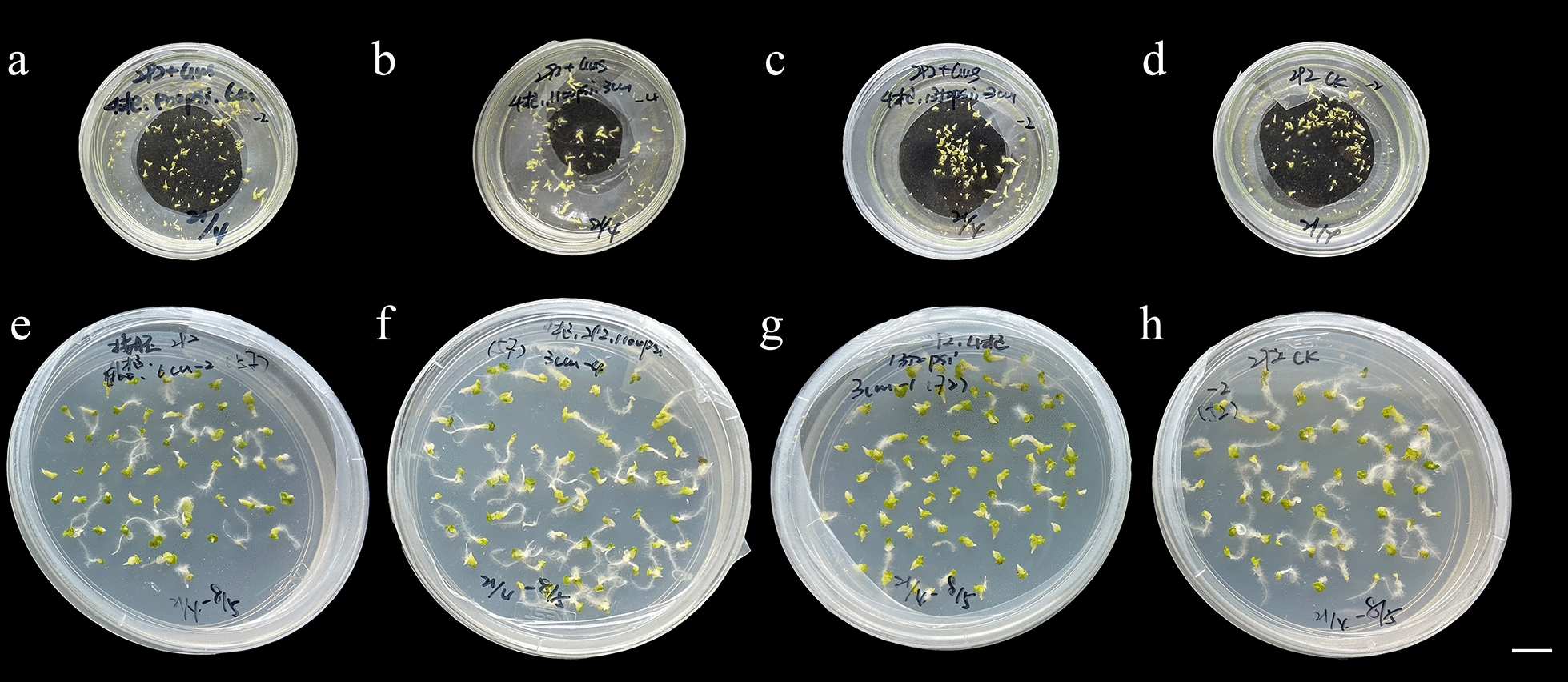
Embryos generated from Chinese cabbage microspores. **a**–**c** Embryos generated from microspores that were treated using the particle bombardment method. **d** The microspores that were not shot (CK). **e**–**g** Embryos from microspores after particle bombardment subcultured in B5 medium; **h** CK. Scale bar = 1 cm

After 15 days, embryoid bodies emerged, and the embryos that developed after the bombardment of the microspores were not significantly different from the embryoid bodies produced by the control (Fig. 4a–h). In conclusion, the particle bombardment treatment had no significant effect on microspore viability or the number of embryos, and particle bombardment could be used as a method to transform the explants' microspores.

**Fig. 5:**
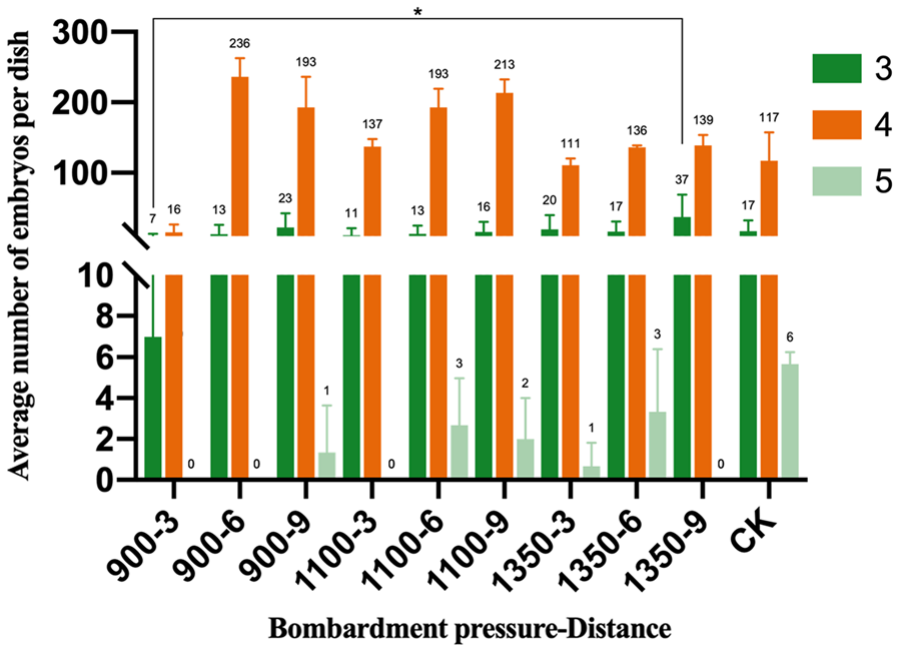
Number of embryos from Chinese cabbage microspores treated by particle bombardment. The green line represents the number of embryos shot 3 times with different pressures and distances, and not shot (CK, control check). The orange lines represent the number of embryos shot 4 times with different pressures and distances, and not shot (CK, control check). The light green lines represent the number of embryos shot 5 times with different pressures and distances, and not shot (CK, control check)

As shown in Fig. 5, except for settings 3 times—900 psi—3 cm and 3 times—1350 psi—9 cm, under the same bombardment times, the number of embryos produced by the bombardment of microspores with different parameters was not significantly different from the control. Under different bombardment times, due to the different bombarded microspore materials and experimental periods, the number of microspore embryos produced in each dish differed.

### The GUS staining of the microspores and embryos

**Fig. 6:**
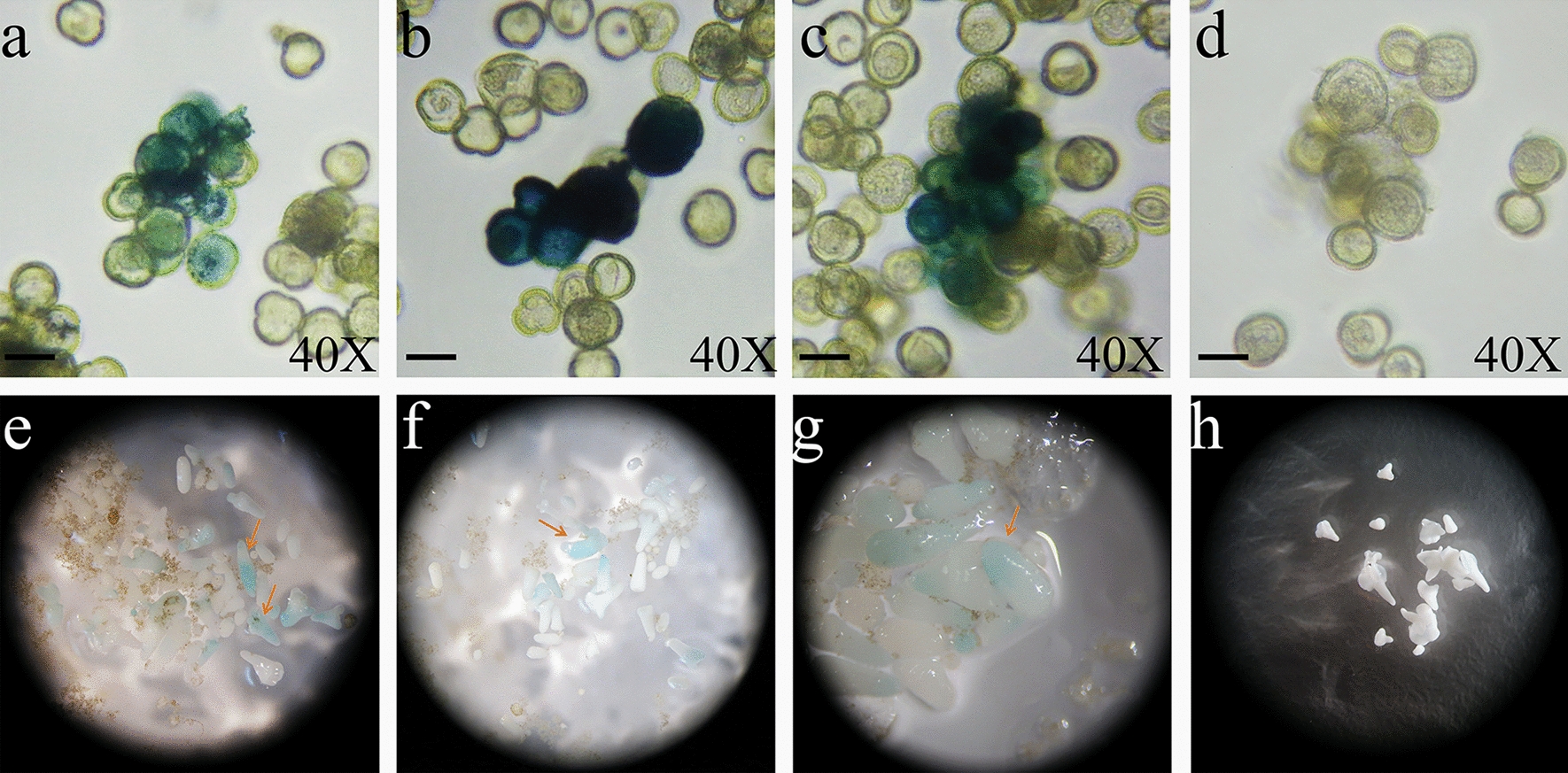
Transformed Chinese cabbage microspores and embryos with a GUS blue color. **a** Transformed microspores using the 4 times—900 psi—3 cm parameter; **b** Transformed microspores using 4 times—1100 psi—3 cm; **c** Transformed microspores using 4 times—1350 psi—3 cm; **d** CK microspores without transformation; **e** Transformed embryos using 4 times-900 psi-6 cm; **f** Transformed embryos using 4 times—900 psi—9 cm; **g** Transformed embryos using 4 times—1350 psi—9 cm; **h** CK embryos without transformation. Scale bar = 20 μm

Using the particle bombardment method, the bombarded and non-bombarded Chinese cabbage microspores were collected and treated with GUS staining solution for 12–24 h, as shown in Fig. 6a–d. The microspores transformed using the particle bombardment method were blue, and the CK microspores were not blue. As a physical transformation method, the biolistic bombardment method realized the instantaneous transformation of Chinese cabbage microspores. After 15 d of cultivation, the embryos were collected and dyed using a GUS staining solution for 12–24 h. Some embryos were blue because they were generated from the shot microspores, and the GUS label was stably inserted into the genome of the Chinese cabbage (Fig. 6e–h).

### Transformation efficiency of the particle bombardment method

After 72 h of cultivation of transformed Chinese cabbage microspores, the number of transformed microspores that were blue was counted, as shown in Table 3. When the bombardment parameters were 4 times–900 psi—3 cm, 4 times–1100 psi—3 cm, and 4 times—1350 psi—3 cm, the number of transformed microspores were 11.67, 11.67, and 21.67 per dish, respectively. There were more transformed microspores than the other parameters. Based on the microspore viability after 72 h, the microspore viability was better when the bombardment parameters were 4 times–1100 psi–3 cm and 4 times—900 psi—3 cm. As a physical transformation method, exogenous GUS plasmid combined with gold particles can be transmitted into microspores and expressed. As shown in Fig. 6e–h, GUS histochemical staining was performed on the embryos induced by the microspores of Chinese cabbage. The number of embryoid bodies was calculated, as shown in Table 3. When the bombardment parameters were 4 times—900 psi—6 cm, the transformation efficiency reached 10.82%, which was proper for the genetic transformation of Chinese cabbage microspores.

**Table 3:** Transformation efficiency of microspores after particle bombardment

Bombardment times	Pressure (psi)	Distance (cm)	Transformed microspores per dish	Transformed embryos	The total number of embryos	Transformation efficiency %
3	900	3	0.00	0	7	0.00
		6	0.00	0	13	0.00
		9	0.67	0	23	0.00
	1100	3	10.00	0	11	0.00
		6	0.00	0	13	0.00
		9	3.33	0	16	0.00
	1350	3	1.67	0	20	0.00
		6	0.00	0	17	0.00
		9	0.00	0	37	0.00
4	900	3	11.67	0	12	0.00
		6	4.67	25	231	10.82
		9	0.00	13	211	6.16
	1100	3	11.67	0	149	0.00
		6	0.67	11	194	5.67
		9	0.00	7	229	3.06
	1350	3	21.67	0	120	0.00
		6	1.67	0	139	0.00
		9	0.00	14	133	10.53
5	900	3	0.00	0	0	0.00
		6	0.00	0	0	0.00
		9	0.00	0	1	0.00
	1100	3	1.33	0	0	0.00
		6	0.00	0	3	0.00
		9	0.00	0	2	0.00
	1350	3	0.00	0	1	0.00
		6	0.00	0	3	0.00
		9	0.00	0	0	0.00

### Identification of transformed plants of T0 generation

**Fig. 7:**
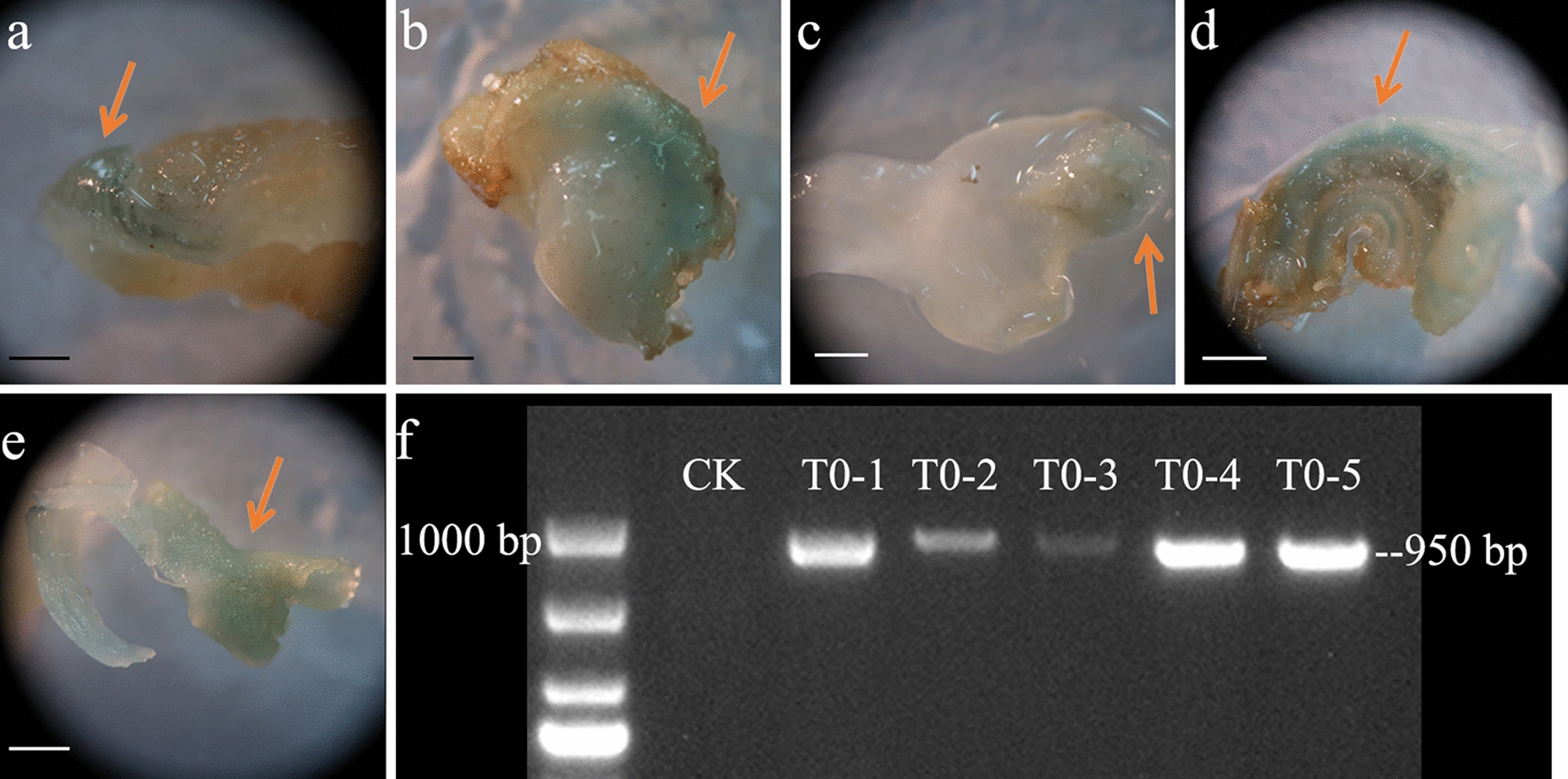
Identification of transgenic plants. **a**–**e** Identification of transgenic plant leaves or tissue by GUS staining, sites that were successfully stained with GUS are shown in blue and marked with arrows; **f** PCR identification of transgenic plants. Scale bar = 1000 μm

GUS staining was performed on the embryos obtained after transformation for 15 days, as shown in Fig. 7a–e. Blue tissue was obtained, indicating that after the microspores were bombarded by the biolistic method, the GUS marker was stably integrated with the development of the microspores. Plant tissue DNA was extracted using the CTAB method, and GUS tag primers were designed for PCR detection. After the test, as shown in Fig. 7f, five plants with the blue GUS staining phenotype and positive bands identified by PCR were obtained. This indicates that the GUS marker was integrated into the T0 generation plants of Chinese cabbage using the particle bombardment method.

**Fig. 8:**
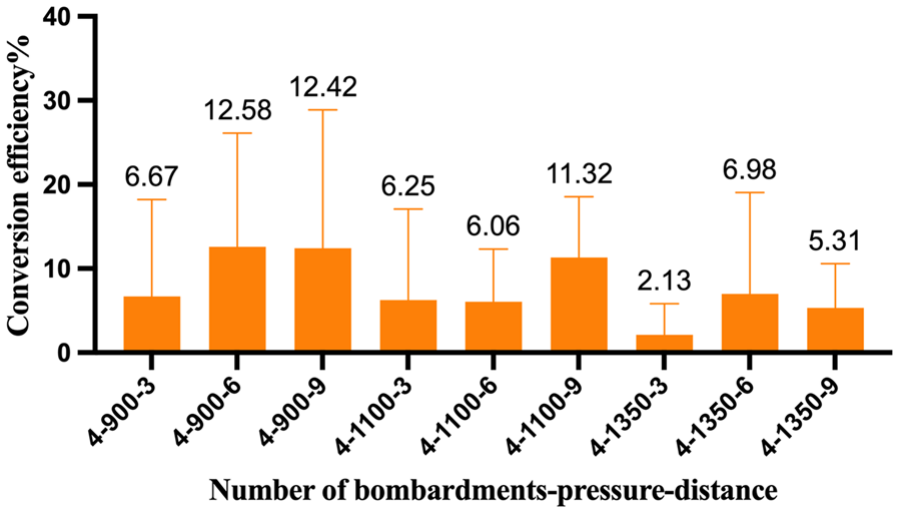
Genetic transformation efficiency of T0 generation plants under different bombardment parameters exhibiting no significant differences between different parameters. Different lowercase letters in the same column indicate significant differences at P < 0.05 based on the LSD test

T0 generation plants were obtained under the condition that the number of bombardment times was 4. The PCR identification results of the samples under different bombardment pressures and bombardment distance combinations were counted, and the statistical results are shown in Fig. 8. The genetic transformation efficiency of Chinese cabbage microspore plants differed. SPSS was employed to detect the significant differences in the conversion efficiency under different parameters, and showed that there was no significant difference between the conversion efficiency of different bombardment pressures and the distance under the condition that the bombardment frequency was 4. Compared with other parameters, there was still a 12.58% chance to obtain transformed plants when the bombardment parameters were 4 times—900 psi—6 cm. Therefore, this parameter was considered appropriate for use in the transformation system.

### Identification of T1 generation transgenic plants

**Fig. 9:**
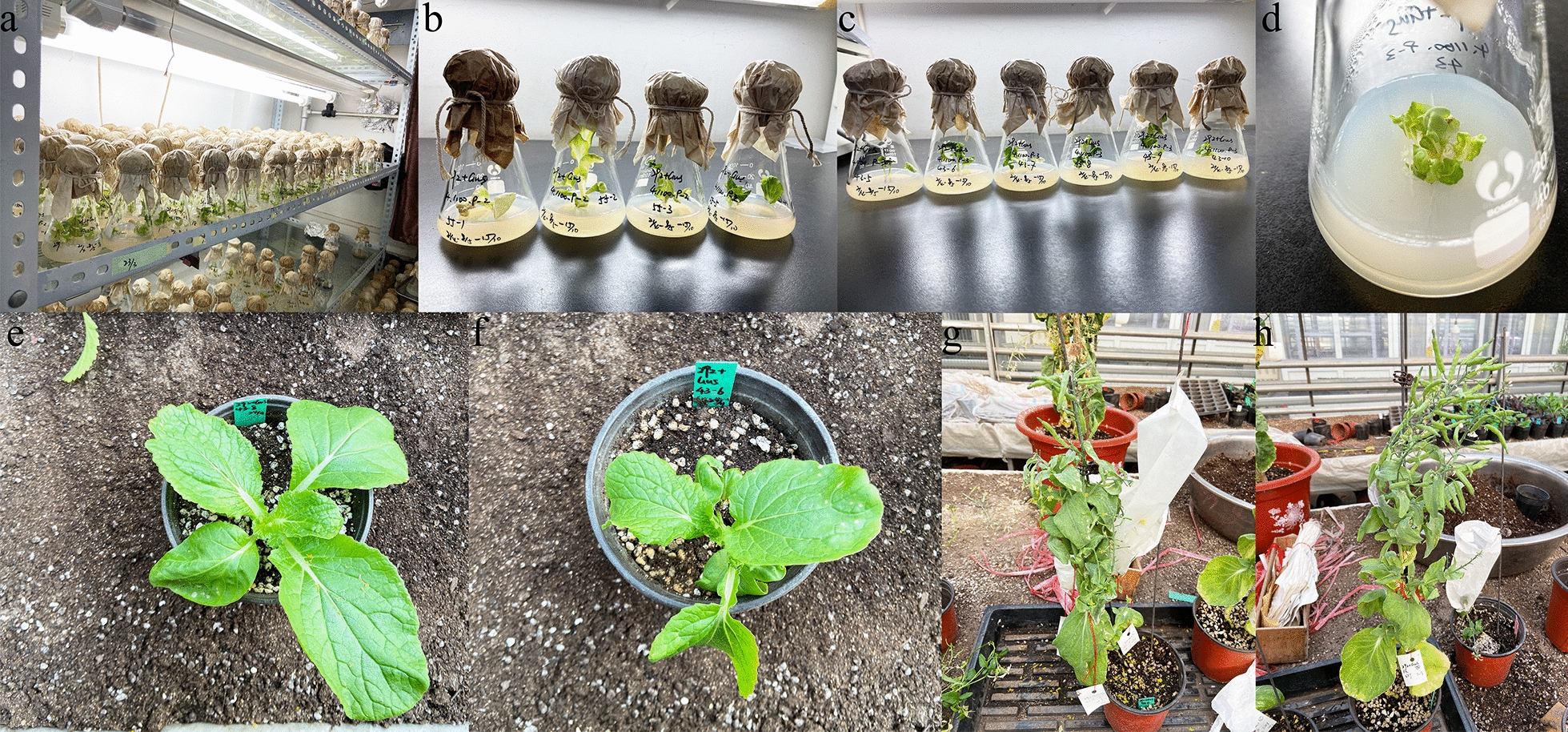
Transformed plants of T0. **a**–**d** Transgenic seedlings at the tissue culture stage. **e**–**f** Transgenic plants were transplanted and cultured in a glass greenhouse. **g**–**h** Transgenic plants were successfully artificially pollinated

As shown in Fig. 9, the screened T0 generation transformed plants were rooted and cultured, and after the newly emerging roots grew, they were transplanted into the seedling matrix for soil culture. The T1 transgenic plants were derived from self-bred offspring of tissue culture seedlings with bombardment parameters of 4 times—1100 psi—9 cm. T1 generation plants were identified using PCR, and the results are shown in Table 4 and Additional file 2: Fig. S2a. A total of 200 (196 survival) T1 generation plants were selected for PCR identification. Among the 196 T1 plants, 15 showed GUS marker-positive fragments, which was inconsistent with the expectation that all T1 plants were transgenic-positive plants. Additionally, 148 (112 surviving) and 190 (172 surviving) T1 generation plants were randomly selected for PCR identification. As shown in Additional file 2: Fig. S2b, c, 10 and 111 plants showed GUS-labeled positive fragments. The genetic transformation efficiency of T1 generation plants is shown in Table 4.

**Table 4:** Genetic transformation efficiency of T1 generation plants

Repeat	Number of positive plants	Total number of plants	T1 Transformation efficiency %
Repeat I	15	196	7.65
Repeat II	10	112	8.93
Repeat III	111	172	64.53

### Particle bombardment-mediated *Dark_Pur* gene transformation in *B. rapa*

**Fig. 10:**
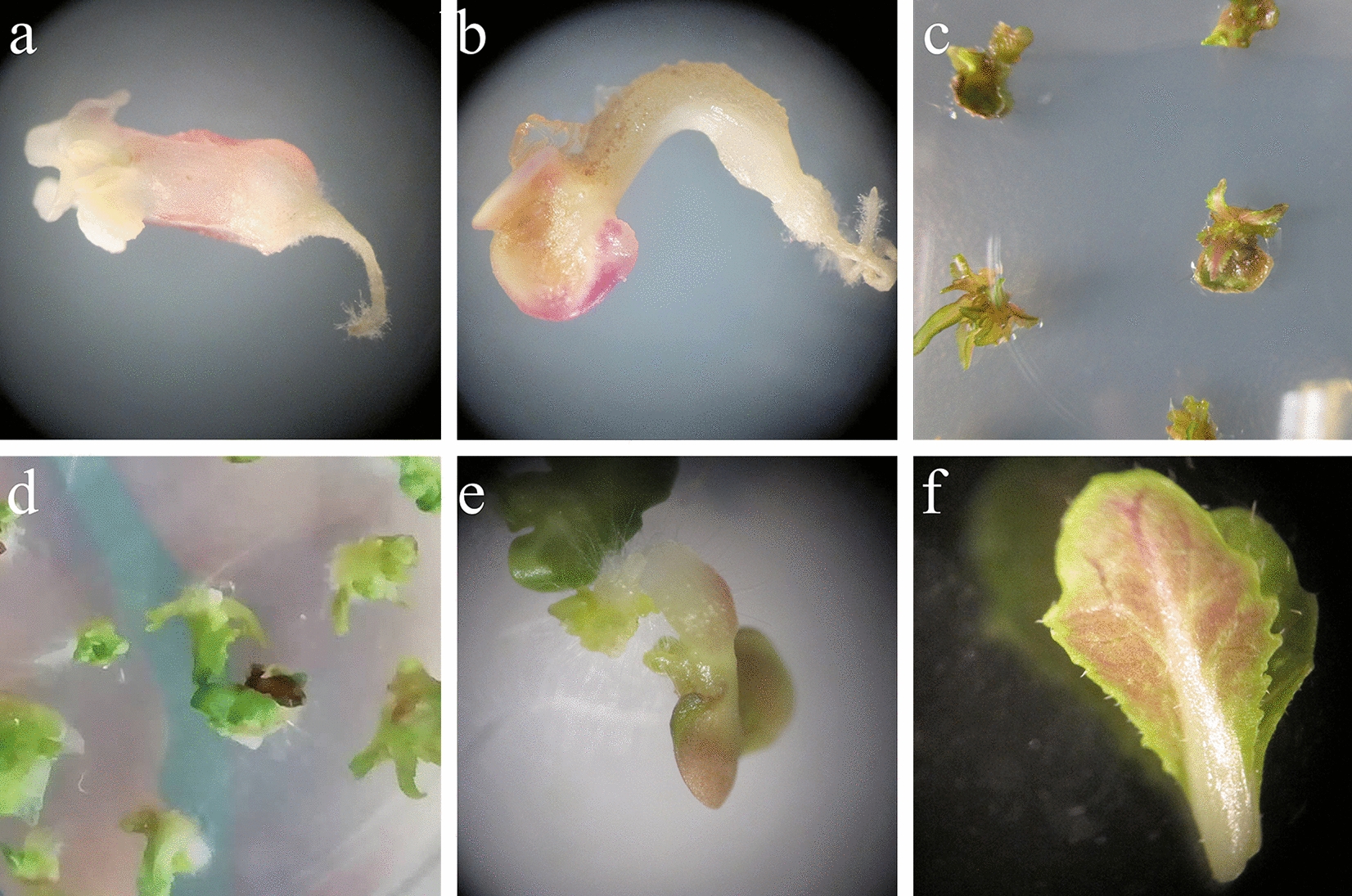
The transformed plants harboring the *Dark_Pur* gene. **a**–**e** Different developmental stages of transgenic purple Chinese cabbage seedlings. **f** Transgenic purple Chinese cabbage leaf

To verify the application of the particle bombardment method, the *Dark_Pur* gene was used as a transformation target. Breeding Chinese cabbage with a purple phenotype is necessary for breeding diversity. Chinese cabbage microspores were used as explants, and the particle bombardment method was used for transformation. Through this transformation method, purple tissue and seedlings were obtained (Fig. 10). This further proved the feasibility of this method and provided gene function verification for the *Dark_Pur* gene.

### Particle bombardment-mediated transformation of mCherry red fluorescent labeling

**Fig. 11:**
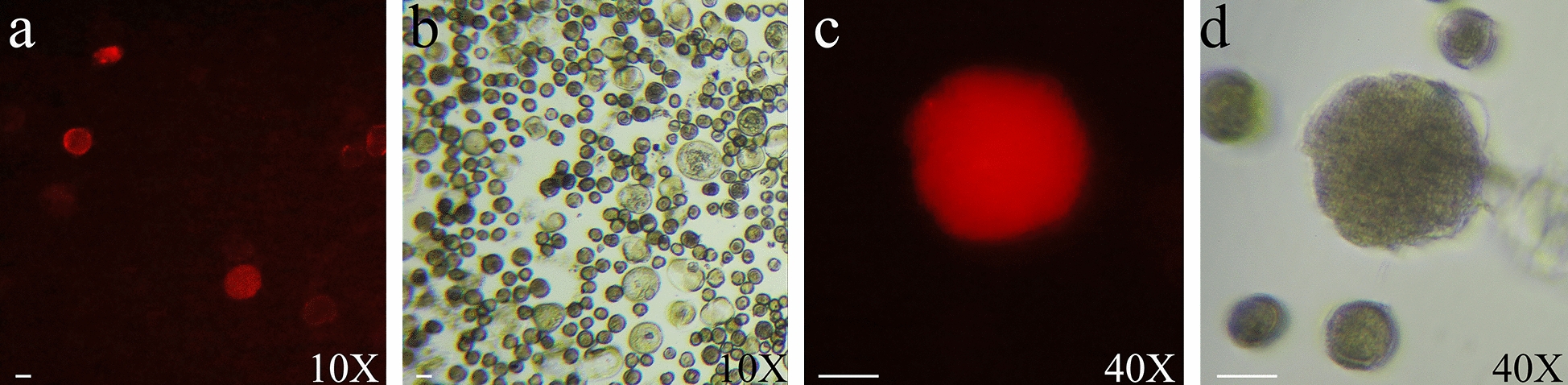
Red fluorescence of Chinese cabbage microspores. **a** Red fluorescence-transformed microspores observed in the 10 × fluorescence field. **b** Corresponding to Chinese cabbage microspores observed in 10X bright field. **c** Red fluorescent cell clusters locked in 40X fluorescence field observation. **d** Corresponding to microspore developmental cell clusters observed in 40 × bright field. Scale bar = 20 μm

A plasmid vector carrying the red fluorescent tag mCherry was constructed and transported into a Chinese cabbage microspore through particle bombardment using the selected proper parameters of 4 times—900 psi—6 cm. The Zeiss inverted fluorescence microscope-Vert.A1 was used to screen microspores showing red fluorescence. As shown in Fig. 11, Chinese cabbage microspores with a red fluorescence phenotype were observed after 72 h of culture, and visible Chinese cabbage microspores expressing red fluorescence developed into cell clusters after 7 days of culture. As a visual selection marker, red fluorescence simplified the screening process for genetically transformed progeny.

## Discussion

### There is little effect of particle bombardment on microspore and embryogenesis viability

One of the key reasons for the difficulty in the genetic transformation of *Brassica* crops is the difficulty in the regeneration of traditional explants because they belong to the difficult regeneration AA genome compared with cabbage-type vegetables (CC genome), which have higher regeneration rates [11]. The key factors affecting the regeneration ability of explants are the genotype of the donor plant and the type of explant. Previous studies on the regeneration ability of different varieties of Chinese cabbage with petioles found that the regeneration ability of different material explants was significantly different. The regeneration frequency of adventitious buds was 9.8–68.2%, and there were differences in the occurrence times of adventitious buds. Some studies have pointed out that the hypocotyl and cotyledon are explant types with higher bud regeneration ability [45, 46], and cotyledon explants have a higher capacity than the hypocotyl [47, 48]. Because the regeneration ability of traditional Chinese cabbage explants is affected by many factors, we propose a new type of genetically transformed explant called microspores and, at the same time, establish a Chinese cabbage genetic transformation system combined with the particle bombardment method. As a new method of genetic transformation, gene guns are widely used in monocotyledonous plants. The most basic requirement for the application of the gene gun-mediated method to the genetic transformation of Chinese cabbage is that there is no significant effect on the vigor of new explants after the bombardment of the gene gun, which can induce the production of regenerated plants. There are a series of factors that influence microspores cultures, and we established a mature microspore culture system based on our former study [44, 49]. In this experiment, the viability of microspores and the ability of microspores to induce embryo regeneration after gene gun bombardment were identified. After inspection, there was no significant difference in the viability of microspores compared with non-bombarded controls under different bombardment parameters. There was no significant difference in the ability of microspores to induce embryoid body regeneration compared to the control. As a new type of genetic transformation receptor, microspores are resistant to gene gun bombardment, thus meeting the requirements of subsequent genetic transformation.

### Proper genetic transformation parameters of the particle bombardment-mediated method

The genetic transformation efficiency of the biolistic method is affected by many factors. The regeneration ability of the donor plant, the concentration of plasmid DNA, the size of the gold particle bullet, and the bombardment parameters of the biolistic method all have an impact on the genetic transformation efficiency of the biolistic method. Previous studies found that the proper bombardment parameters for different materials differed. For the genetic transformation of grape embryo suspension cells, the transient expression efficiency was better when the helium bombardment pressure was 1000 or 1200 psi [50]; In tobacco, genetic transformation was performed, and the transformation efficiency was higher when the bombardment pressure was 200–250 psi [51]; Genetic transformation of mouse brain tissue was carried out, and bombardment pressure of 50 psi had a better genetic transformation rate [52]. The time of bombardment also affects genetic transformation. Compared with 3 times, when chloroplasts were transformed, 2 biolistic bombardments had the best transformation efficiency [51].

In this study, the bombardment parameters of the gene gun were screened. The bombardment times were 3, 4, and 5, the bombardment pressures were 900, 1100, and 1350 psi, and the bombardment distances were 3, 6, and 9 cm. The three parameters were combined to determine the proper particle bombardment parameters. When the bombardment parameters of the gene gun were 4 times—1350 psi—3 cm, 4 times—1100 psi—3 cm, and 4 times—900 psi—3 cm, the highest transient expression efficiency was obtained, and the average number of transformed microspores per dish was 21.67, 11.67, and 11.67, respectively. When the number of bombardment parameters was 4 times—900 psi—6 cm, the proper genetic transformation efficiency was 10.82%.

### Analysis of the integration stability of exogenous GUS markers in the T0 and T1 generations

After culturing the microspore-induced regeneration plants, which were dealt with by particle bombardment, 5 transformed plants were obtained, and two self-crossing plants were successfully obtained. After random seeding identification, the plants with GUS fragments in the T1 generation accounted for 7.65–64.53% of all identified plants. Because the transformed explants were microspores of Chinese cabbage at the stage of late uninucleate stage microspores, the T0 generation plants should be harvested as DH lines. The identified transgenic generation plants obtained from T0 self-crossing should all be transgenic individual plants, but the PCR identification results were inconsistent with the theory.

The method of obtaining Chinese cabbage microspores was to extract microspores from Chinese cabbage buds with a diameter of 2–3 mm. If the number of microspores is large, there may be binucleate-stage microspores mixed in. If the microspore source of the regenerated plant is induced by a gene gun, the regenerated progeny may have a trait separation. Although the particle bombardment method has almost no restrictions on the explants, it is prone to a multi-copy phenomenon during the transformation process, and the offspring will have a certain degree of gene segregation and silencing. This may be one reason why a 3:1 separation ratio was not obtained for T1 progeny [53]. Solving the defect of trait segregation and silencing in the progeny of particle bombardment transformation is a key issue for further application of the gene gun method.

### mCherry red fluorescent labeling application

Because GUS markers have lethal defects in histochemical staining, selecting a vector with fluorescent selection markers for genetic transformation can avoid lethal defects in GUS marker selection. Red fluorescent protein (DsRed) can be extracted from coral, has an absorption maximum of 583 nm, can be detected without any pretreatment, and is widely used [54]. Because of its stable expression and non-toxicity to cells, red fluorescence is widely used in visual identification. Tobacco protoplasts were transformed with a vector containing red fluorescent tags to achieve the transient expression of DsRed [55]. It has been widely used as a reporter gene [56–58]. In addition to red fluorescence, green fluorescence is used as a screening tag [59]. GFP is widely used as a screening tag in animals, plants, and microorganisms [60–62].

In this experiment, with the help of the constructed mCherry vector containing a red fluorescent tag, the genetic transformation of Chinese cabbage microspores was carried out by particle bombardment, and red fluorescent cells and cell clusters were successfully obtained, which proved that red fluorescence can be used as a screening tag in the early stage of the genetic transformation of Chinese cabbage microspores. However, red fluorescent embryos and plants have not been obtained, and further research is needed to improve the application of red fluorescence as a screening marker in the genetic transformation of Chinese cabbage microspores.

The transformation of Chinese cabbage is generally difficult (transformation rate: < 1%) [63]. Using the *Agrobacterium*-mediated transformation method, obtaining transgenic plants usually requires 3–4 months. Compared to the newly published method with a transformation efficiency of 10.83%, the particle bombardment method established in this research has the nearly same efficiency, and the regeneration time is greatly reduced due to the use of the new explant, which is a germ cell instead of a somatic cell [15]. Direct gene transfer by particle bombardment to microspores greatly overcomes the difficulty of tissue regeneration of explants, and after two weeks of bombardment, transgenic embryos can be selected. The gene gun  established here can effectively shorten the time required to obtain transgenic Chinese cabbage plants. In this study, we established an efficient and stable genetic transformation system that will promote studies on gene function and trait improvement in Chinese cabbage.

### Supplementary Information

**Additional file 1. **Viability of microspores after particle bombardment (72 h) observed using a microscope at 20X. The numbers 1–27 represent the different parameters of particle bombardment, and the parameters are the same as those given in Table 2. CK-1 represents CK viability in the same sampling period as 1–9, CK-2 represents CK viability in the same sampling period as 10–18, and CK-3 represents CK viability in the same sampling period as 19–27.

**Additional file 2. **PCR identification of T1 generation. a-c Repeated I to repeat III.

## Data Availability

Data available on request from the authors.
