# Supplementary figures and images for: Efficient transformation of the isolated microspores of Chinese cabbage (Brassica rapa L. ssp. pekinensis) by particle bombardment
Source: Plant Methods. 2024 Jan 30;20:17. doi: 10.1186/s13007-024-01134-1 (PMC10826076; doi:10.1186/s13007-024-01134-1)

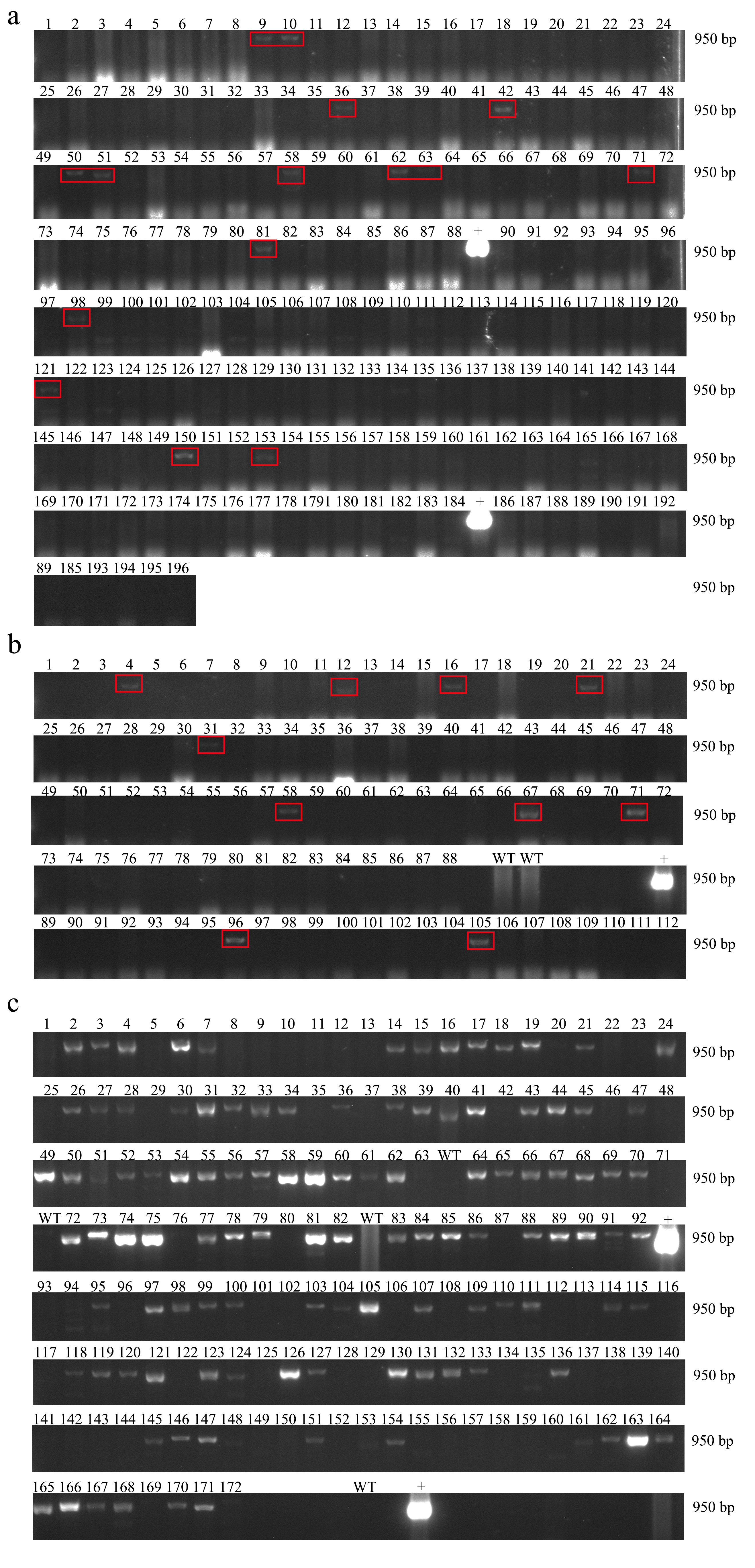

Supplement: Supplementary file 2 — Additional file 2. PCR identification of T1 generation. a-c Repeated I to repeat III. [file 13007_2024_1134_MOESM2_ESM.png]
